# Association Between Health Literacy and Work Ability in Employees With Health-Related Risk Factors: A Structural Model

**DOI:** 10.3389/fpubh.2022.804390

**Published:** 2022-02-18

**Authors:** Madeleine Gernert, Gerrit Stassen, Andrea Schaller

**Affiliations:** Working Group Physical Activity-Related Prevention Research, Institute of Movement Therapy and Movement-Oriented Prevention and Rehabilitation, German Sport University Cologne, Cologne, Germany

**Keywords:** health promotion, health literacy, work ability, structural model, PLS-SEM (partial least squares structural equation modeling)

## Abstract

**Introduction:**

In workplace health promotion (WHP), health literacy and work ability are considered as outcomes of high interest. Therefore, the question arises as to what extent individual health literacy skills have an impact on work ability alongside sociodemographic influences.

**Objectives:**

This study aimed to examine the associations between a structural model of health literacy as well as sociodemographic context factors and the work ability among employees with health-related risk factors.

**Materials and Methods:**

The study was based on baseline data of a workplace-related intervention (158 employees with health-related risk factors, 53.8% women, 48 ± 10 years). Health literacy skills were assessed with Lenartz's Questionnaire (measuring “self-perception”, “proactive approach to health”, “dealing with health information”, “self-control”, “self-regulation”, and “communication and cooperation”). Work ability was measured by the German Short Form of the Work Ability Index (WAI). As sociodemographic context factors, sex, age, and educational level were assessed. The associations were examined using structural equation modeling with partial least squares (SmartPLS 2.0.M3). Common quality criteria were applied and significance level was set at α = 5%.

**Results:**

Model's reliability, validity, and structure could be validated. Regarding the impact on work ability, “self-regulation” showed a statistically significant direct effect (ß = 0.32, *t*_(∞)_ = 4.00, *p* < 0.01, *f*^2^ = 0.09) and “self-perception” had a significant indirect effect (ß = 0.13, *t*_(∞)_ = 2.53, *p* < 0.05). The only additional association with work ability was found for age (ß = −0.25, *t*_(∞)_ = 3.82, *p* < 0.01, *f*^2^ = 0.04). The WAI score variance was explained to 17.5% by the health literacy skills and to 27.5% considering the additional sociodemographic context factors.

**Conclusion:**

According to the structural model of health literacy, in employees with health-related risk factors, a target group-specific WHP approach could be the encouragement of self-regulation and self-perception. However, additional resources and conditions influencing work ability should be considered.

## Introduction

In Germany, almost one third of the adult population rate their general health as fair, poor, or very poor (1) and even about half of the population has a chronic disease or long-term health problem (2). The most prevalent health impairments are related to internal cardiometabolic (e. g., hypertension, hyperlipidemia, obesity) and musculoskeletal (e.g., chronic back pain, osteoarthritis) conditions (3). For example, in the target group of employees, 41% show the cardiovascular risk factor hypertension (4), and 70% report at least one musculoskeletal complaint (5). Additionally, mental health impairments like physical or emotional exhaustion and fatigue are increasing (5). As a result, primary and secondary prevention activities to prevent diseases or health problems, reduce the risk of disease or delay the onset of the same, are emphasized (6).

One key determinant of health today is considered to be health literacy (7), which as a concept is integrally linked to the field of health promotion (8–10). Health promotion is defined as the “process of enabling people to increase control over, and to improve, their health”, including physical, mental, and social well-being (11). Accordingly, the concept of “health literacy is linked to literacy and entails people's knowledge, motivation and competences to access, understand, appraise and apply health information in order to make judgements and take decisions in everyday life concerning health care, disease prevention and health promotion to maintain or improve quality of life during the life course” (7, 12). In this regard, studies have confirmed the association of health literacy with health status, health behavior, and health risk factors (13–16). Yet, it is particularly people with health problems who need a high level of health literacy since they have to take more responsibility for their health (17). However, a variety of different concepts and definitions of health literacy emerged to date (18), which is why it is essential to refer to specific models in health literacy studies.

**Figure 1:**
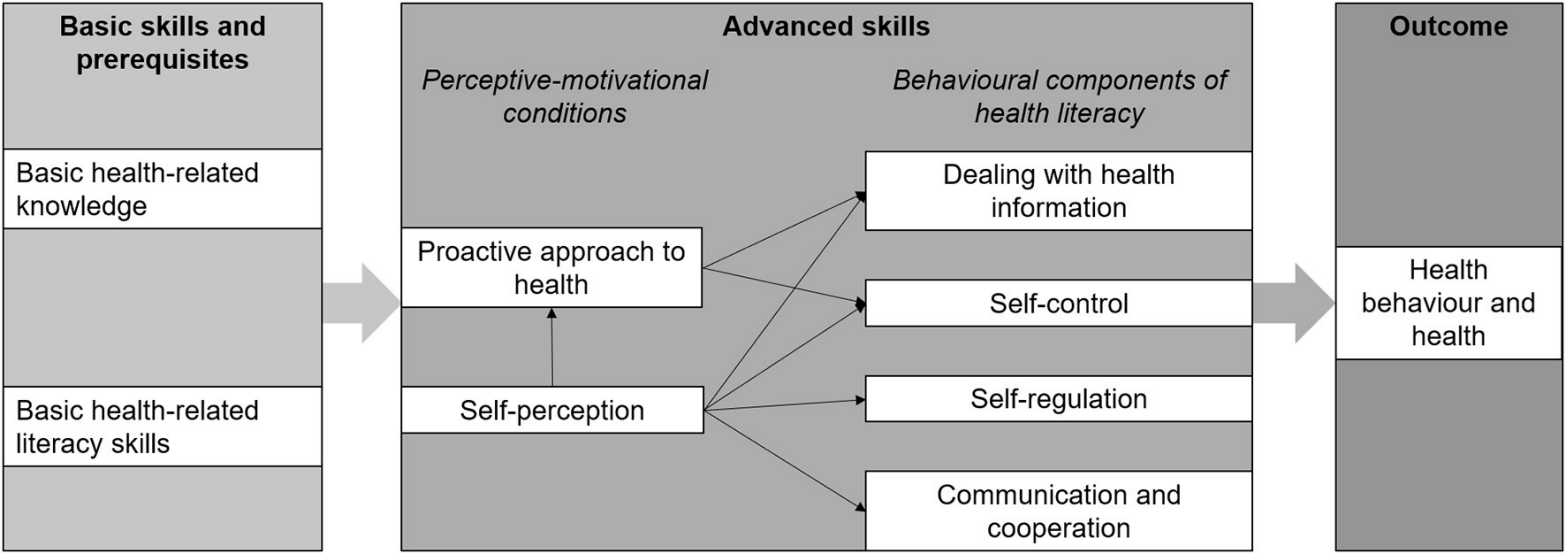
Structural model of health literacy according to Lenartz (19) and Soellner et al. (20).

Against this background, Lenartz developed a structural model of health literacy (Figure 1) with the intention to contribute to the content development of health promotion interventions in different contexts (19, 20). The model consists of basic health-related knowledge and literacy skills as well as six advanced health literacy skills (perceptive-motivational conditions and behavioral components of health literacy) explaining health status and health behavior through their indirect and direct influence (19–21). So far, the included health literacy skills were associated with physical and mental health as well as health behavior in the target groups of pupils and adults (19), with the absence of physical complaints in students (22), with psychological well-being in adults (23), and with work ability in vocational school students (24).

Another target group with increased interest in health promotion are employees with health-related risk factors, as they seem to be vulnerable in terms of both health literacy and work ability. Since health literacy is an individual resource for health (13–16), which in turn has a significant influence on work ability (25, 26), the question arises as to what extent health literacy skills have an impact on work ability in this target group. Nevertheless, sociodemographic context factors should also be considered as they are known to have a relevant effect on health status and health literacy (27, 28).

The present study examined a structural health literacy model in the target group of employees with health-related risk factors. Research questions were:

(1) Can the structural model of health literacy be validated in a sample of employees with health-related risk factors?
(2) To what extent are the advanced skills within the structural model of health literacy associated with the target group's work ability?
(3) To what extent are sociodemographic context factors additionally associated with the target group's work ability?

## Materials and Methods

### Study Design and Data Sources

A secondary analysis of baseline data of the AtRisk study was conducted. The subject of the AtRisk study was the evaluation of a cross-provider workplace-related health promotion intervention for employees with health-related risk factors (29). Within the AtRisk study, this target group was defined as employees with initial health impairments which do not yet indicate rehabilitation, but which probably have an unfavorable influence on the individual's work ability. This health condition was described by the term “employees with health-related risk factors”. Following a company doctor's medical entry examination, potential participants were invited to the behavior-related secondary prevention intervention. Eligibility criteria were (1) a formally approved application for a preventive health service by the German Pension Fund, (2) age 18 to 65 years, (3) first health impairments (of the musculoskeletal system, internal organs or mental impairments) understood as health-related risk factors, and (4) written informed consent to participate in the study (29). Exclusion criteria were (1) the indication for a rehabilitative treatment, (2) the need for acute care, and (3) lack of understanding the German language (29). Baseline data were collected by self-reporting paper-pencil questionnaires between July 2016 and August 2017. Ethical approval was provided by the German Sport University Cologne Ethics Committee (reference number 93/2015).

### Measures

In order to assess health literacy, Lenartz's German health literacy questionnaire (19) was used. It is based on the structural model of health literacy (Figure 1) and consists of 29 items to be appraised on a four-point scale (1 = “not correct at all”, 2 = “rather not correct”, 3 = “rather correct”, 4 = “correct”) depicting the six advanced skills. For the six subscales, mean values are calculated. The questionnaire has been applied in different target groups and validated with multiple outcomes (19, 22–24).

To assess work ability, the German short-form of the work ability index (WAI) (30) which is recommended for group surveys in the setting of workplace health promotion (WHP) (31) was applied. Ten items on the demands of work, employee's health status, and resources in the context of work are assigned to seven dimensions and standardized point values are given for each answer (30). According to the resulting sum score between 7 and 49 points, work ability is considered to be poor (7–27), moderate (28–36), good (37–43), or very good (44–49) (30). Validity (25, 32–35) and reliability (32, 35, 36) were confirmed repeatedly.

Additionally, participants' sex (male/female), age (years), and the highest level of educational level were determined as sociodemographic context factors. Educational level was described as low (no general school certificate or general school certificate without university entrance qualification) medium (general school certificate with university entrance qualification) or high (university degree) based on the highest level of education.

### Statistical Analyses

In the present secondary analysis, incomplete questionnaires were not considered. It was examined whether missing values were missing completely at random (MCAR) (37).

To describe the sample, descriptive statistics (means, SDs, minima, maxima, 95% confidence intervals, frequencies) were calculated.

To test the model structure and to examine the associations between the advanced health literacy skills, sociodemographic context factors, and work ability, structural equation modeling (SEM) with partial least squares (PLS) was conducted with SmartPLS 2.0.M3 (38). PLS-SEM is a non-parametric method which also works with binary coded variables and should be chosen if the primary objective of applying structural modeling is prediction and explanation of target constructs (39). In PLS-SEM, relations between latent and manifest variables (measure/outer model) as well as between latent variables (structural/inner model) are defined in form of path models. The directional interpreted paths are represented by connecting arrows (40).

To validate Lenartz's structural health literacy model (see research question 1), the inner model was built of the six advanced skills according to the model structure (Figure 1). “Self-perception” is the only independent (exogenous) latent variable with all other dimensions being dependent (endogenous) latent variables (“proactive approach to health”, “dealing with health information”, “self-control”, “self-regulation”, “communication and cooperation”) (19). For each subscale, items were summarized into parcels in order to ensure comparability with previous studies (19, 22, 24). The item parcels served as reflective indicators for the related latent constructs (outer model). To assess internal consistency reliability, Cronbach's α and composite reliability were both compared to the benchmark >0.7) (40). Convergent validity was assessed by the indicators' significant outer loadings (benchmark >0.7, *p* < 0.05) and the average variance extracted (AVE) for each latent variable (benchmark >0.5) (40). Discriminant validity was considered if the indicators correlated highest with the related construct (cross-loadings) (40). Additionally, the Fornell-Larcker criterion, stating that the square roots for each latent variable's AVE should be higher than its highest correlation with any other variable, was applied (41).

The structural model's predictive power was evaluated by the determination coefficient *R*^2^ and its effect size *f*^2^. *R*^2^ indicates the proportion of the variance of the endogenous constructs that is described by all associated latent variables (40) with *R*^2^> 0.02 being considered a small, *R*^2^> 0.13 a median and *R*^2^> 0.26 a large effect in the behavioral sciences (42). The effect size *f*^2^ describes the influential amount of a latent variable on the variance explained (*f*^2^> 0.02 small, *f*^2^> 0.15 medium, *f*^2^> 0.35 large effect) (42).

The significance of the paths was estimated by bootstrapping processes (158 cases, 5,000 samples, df = ∞) using critical *t*-values of >1.960 (*p* < 0.05) and >2.576 (*p* < 0.01) (40, 43).

To examine the associations between health literacy skills and work ability (see research question 2), the WAI sum score was included as a further endogenous variable and connected with the four behavioral components of health literacy.

Additionally, the independent variables sex, age, and educational status were linked to work ability, to examine the direct relationship of these sociodemographic context factors with work ability (see research question 3).

As a result, the highest number of direct paths to the construct of work ability is seven (four behavioral components of health literacy and three sociodemographic context factors) which is multiplied by 10 to calculate the necessary sample size (70 cases) according to a common rule of thumb in the methods of structural equation modeling (44).

## Results

### Sample and Descriptive Results

Two hundred fifty-six employees with health-related risk factors took part in the baseline survey of the underlying AtRisk study. One fifty-eight participants (61.7%) provided complete baseline data, thus the required sample size has been reached (70 cases). Incomplete answers were missing completely at random (MCAR). As a result, 158 respondents (53.8% female, 48 ± 10 years) were included in the following analysis (Table 1).

**Table 1:** Sample characteristics.

**Categorial sample characteristics** (***n*** **= 158)**		***n***	**%**	**95%-CI**
Sex (female)		85	53.8	47.2–60.4
**Educational level**				
Low		80	50.8	44–57.2
Medium		48	30.4	24.5–36.4
High		30	19	13.9–24.1
**Work ability status**				
Poor		11	7	3.7–10.3
Moderate		56	35.4	29.4–41.4
Good		79	59	43.6–56.4
Very good		12	7.6	4.2–11.1
**Metric sample characteristics**		**M** **±SD**	**MIN; MAX**	**95%-CI**
Age (years)		48 ± 10	20; 63	45.9–49.2
Health literacy (scale: 1–4)				
Perceptive-motivational conditions	Self-perception	2.9 ± 0.4	2.0; 4.0	2.8–3.0
	Proactive approach to health	2.6 ± 0.5	1.4; 4.0	2.6–2.7
Behavioral components of health literacy	Dealing with health information	3.0 ± 0.5	1.6; 4.0	2.9–3.1
	Self-control	2.9 ± 0.4	1.6; 4.0	2.8–2.9
	Self-regulation	2.4 ± 0.6	1.0; 4.0	2.3–2.5
	Communication and cooperation	2.5 ± 0.6	1.3; 4.0	2.4–2.6
Work ability score (WAI) (scale: 7–49)		36.4 ± 5.3	22.5; 47.5	35.5–37.2

*M, mean value; SD, standard deviation; MIN, minimum; MAX, maximum; 95%-CI, 95% confidence interval; WAI, work ability index*.

### Measure Model

Cronbach's α was >0.7 except for self-perception (α = 0.69) and composite reliability (CR) was >0.7 for all variables (Table 2). Each variable had an AVE >0.5 (Table 2) and significant outer loadings >0.7 (Table 3). All indicators correlated highest with their related construct (Table 3) and the Fornell-Larcker criterion (Table 2) was fulfilled.

**Table 2:** Indicators for internal consistency reliability, convergent and discriminant validity of the structural health literacy model.

**Dimension**	**Cronbach‘s** ***α***	**CR**	**AVE**	**Fornell-Larcker criterion**					
				**SP**	**PAH**	**DHI**	**SC**	**SR**	**CC**
Self-perception	0.69	0.86	0.76	**0.87**					
Proactive approach to health	0.79	0.91	0.83	0.40	**0.91**				
Dealing with health information	0.84	0.92	0.86	0.33	0.32	**0.93**			
Self-control	0.77	0.89	0.81	0.44	0.32	0.26	**0.90**		
Self-regulation	0.74	0.89	0.79	0.18	0.19	0.16	0.35	**0.89**	
Communication and cooperation	0.81	0.91	0.84	0.33	0.36	0.24	0.27	0.42	**0.92**

*CR, composite reliability; AVE, average variance extracted; bold, AVE's square roots; SP, self-perception; PAH, proactive approach to health; DHI, dealing with health information; SC, self-control; SR, self-regulation; CC, communication and cooperation*.

**Table 3:** Cross loadings of item-parcels in Lenartz's health literacy questionnaire as indicator for discriminant validity.

**Item-parcel**	**SP**	**PAH**	**DHI**	**SC**	**SR**	**CC**	**l**	***t*** **(df = ∞)**
SP A	**0.84**	0.29	0.28	0.31	0.10	0.27	0.84^**^	19.10
SP B	**0.91**	0.39	0.30	0.45	0.21	0.31	0.91^**^	42.92
PAH A	0.31	**0.89**	0.25	0.30	0.19	0.32	0.89^**^	28.72
PAH B	0.41	**0.93**	0.32	0.29	0.16	0.35	0.93^**^	56.72
DHI A	0.27	0.33	**0.93**	0.17	0.13	0.31	0.93^**^	53.80
DHI B	0.35	0.26	**0.93**	0.31	0.17	0.13	0.93^**^	52.39
SC A	0.33	0.24	0.26	**0.87**	0.33	0.24	0.87^**^	25.30
SC B	0.45	0.33	0.22	**0.93**	0.30	0.26	0.93^**^	62.51
SR A	0.17	0.14	0.12	0.29	**0.90**	0.34	0.90^**^	5.27
SR B	0.15	0.20	0.17	0.33	**0.88**	0.41	0.88^**^	5.57
CC A	0.30	0.34	0.15	0.27	0.38	**0.92**	0.92^**^	24.61
CC B	0.31	0.33	0.28	0.25	0.39	**0.92**	0.92^**^	29.40

*Bold, highest correlation; l, outer loadings; t, t-value; SP, self-perception; PAH, proactive approach to health; DHI, dealing with health information; SC, self-control; SR, self-regulation; CC, communication and cooperation*.

** *p < 0.01*.

### Structural Models

**Figure 2:**
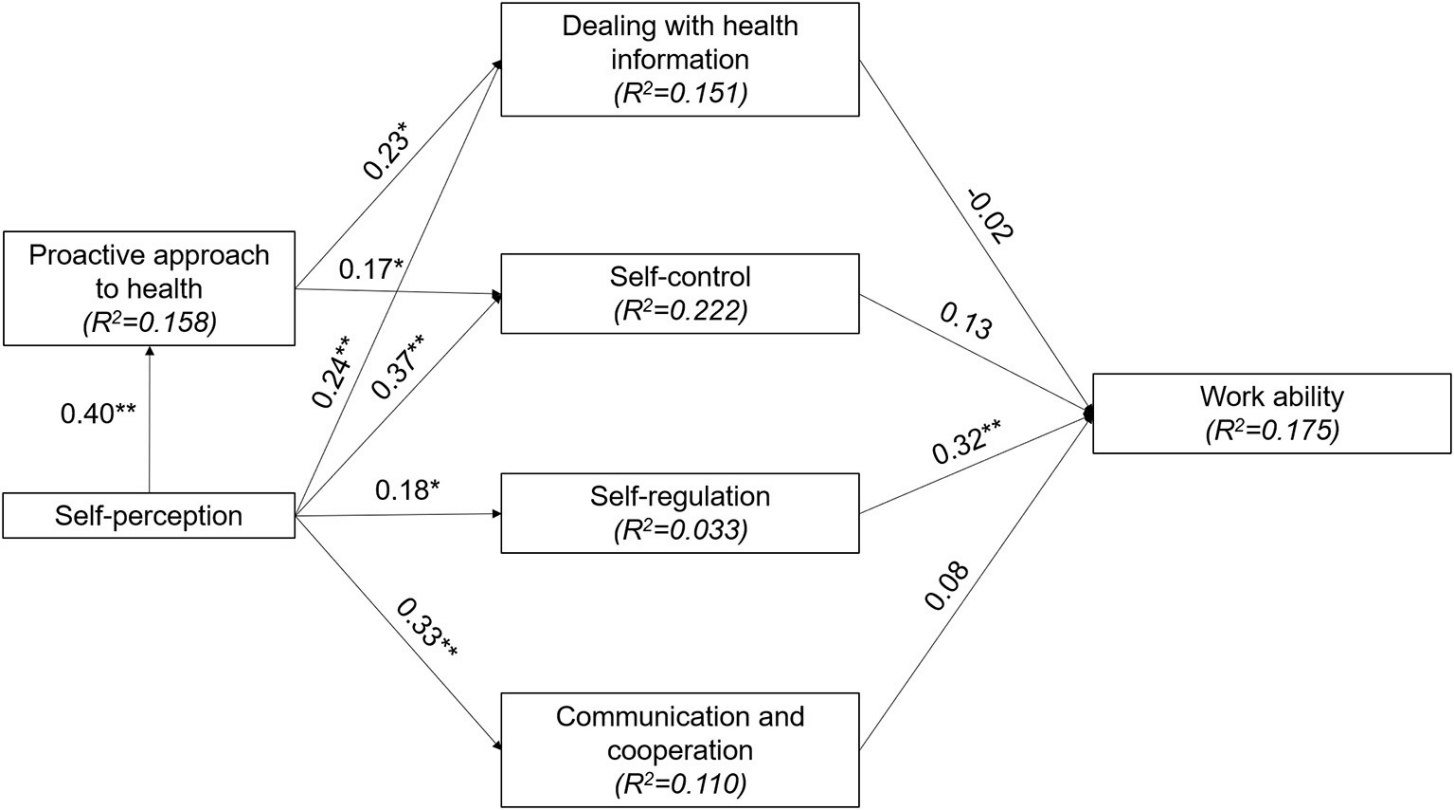
Structural model of health literacy including work ability with determination coefficients (*R*^2^) and path coefficients. *p < 0.05, **p < 0.01.

All path coefficients within the structural model of health literacy were statistically significant (Figure 2). “Self-control” explains the highest proportion of variance (22.2%) within the model. The highest path coefficients lead from “self-perception” to “proactive approach to health” (ß = 0.40) and “self-control” (ß = 0.37).

Work ability's determination coefficient was moderate (17.5%). “Self-regulation” showed the only statistically significant direct effect on work ability (ß = 0.32, *t*_(∞)_ = 4.00, *p* < 0.01, *f*^2^= 0.09). In addition, “self-perception” had a significant indirect effect (ß = 0.13, *t*_(∞)_ = 2.53, *p* < 0.05).

**Figure 3:**
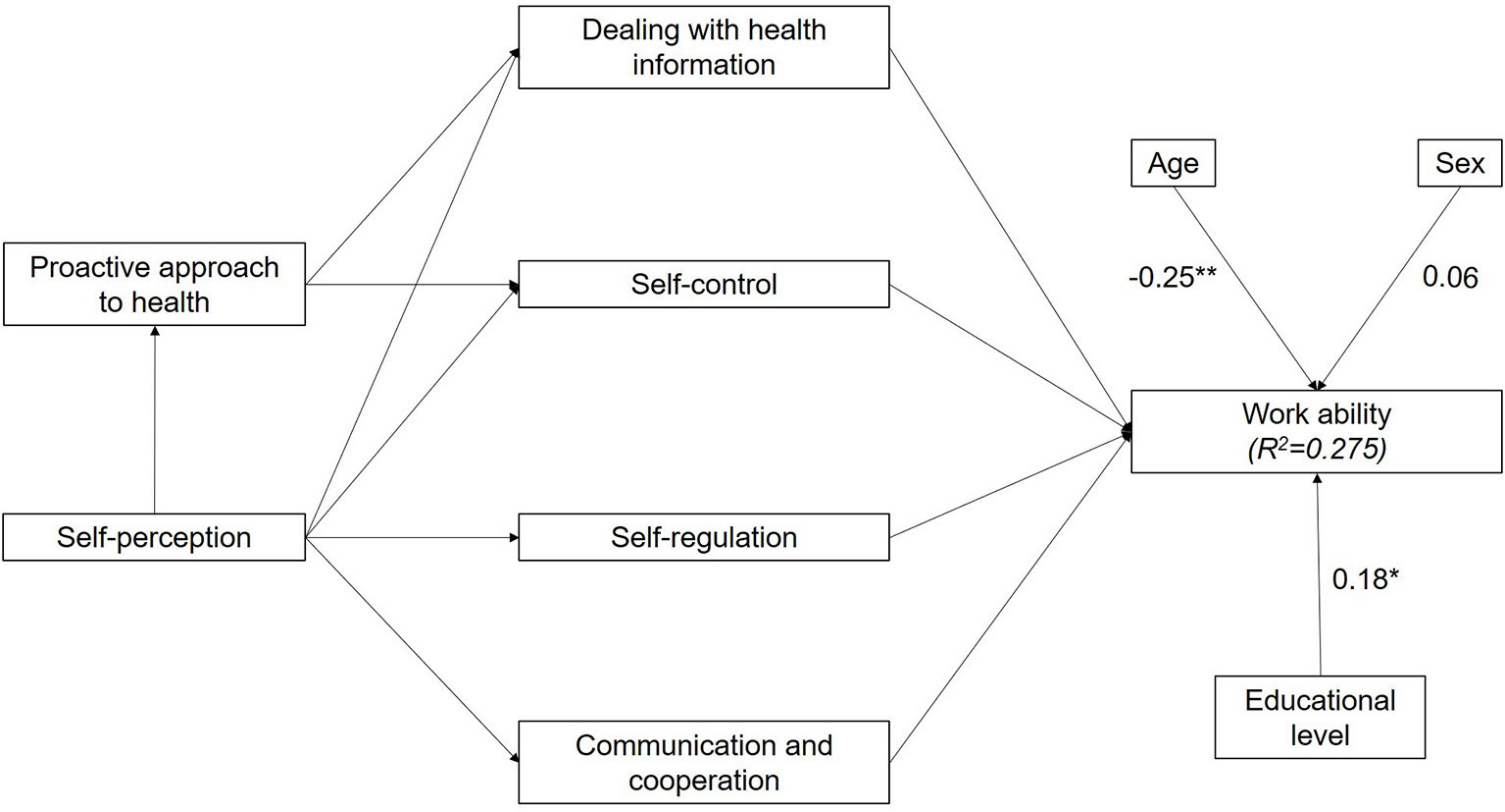
Structural model of health literacy including sociodemographic context factors and work ability with determination coefficients (*R*^2^) and path coefficients. *p < 0.05, **p < 0.01.

After extending the model with sociodemographic context factors, work ability's determination coefficient increased to 27.5%, explaining a large proportion of variance (Figure 3). The strongest additional path coefficient to work ability comes from age (ß = −0.25, *t*_(∞)_ = 3.82, *p* < 0.01, *f*^2^= 0.04), followed by educational level (ß = 0.18, *t*_(∞)_ = 2.54, *p* < 0.05, *f*^2^= 0.01) and sex (ß = 0.06, *t*_(∞)_ = 0.83, *p* > 0.05, *f*^2^= −0.03). As a result, age is the only sociodemographic context with a negative significant and small effect on work ability, since the significant effect of educational level has no relevant effect size and the small effect size of sex is not significant.

## Discussion

Our results showed that Lenartz's structural model of health literacy was valid in the target group of employees with health-related risk factors. Health literacy skills explained 17.5% of the WAI score variance and with the incorporation of sociodemographic context factors, 27.5% of the WAI score variance was explained. Work ability was positively associated with “self-regulation” and “self-perception” and negatively associated with age.

Following the validation of the structural model of health literacy in vocational school students (24), students (22), and IT managers (23), it could also be replicated in the target group of employees with health-related risk factors. Mean health literacy scores vary between 2.4 and 3.0 which is comparable to previous studies (19, 22–24), and the path coefficients within the structural model were significant. However, the path coefficients and proportions of explained variance differed in their values from previous studies, which could indicate target group-specific characteristics of health literacy (19, 23, 24).

Although health literacy is an essential concept in the field of health promotion (8–10), the level of health literacy in the German population is particularly low in terms of prevention and health promotion (27). Additionally, health inequalities are apparent in vulnerable groups, which are more likely to have reduced health literacy (e. g., people with a lower level of education, chronic illness, or long-term health problems) (27), requiring targeted health literacy promotion interventions.

Regarding the influence of the advanced health literacy skills on work ability, “self-regulation” and “self-perception” showed significant associations. Yet, these skills are established concepts in behavioral psychology. In the field of health promotion, self-regulation is considered an important resource for behavioral change (45) and has been associated with nutrition and exercise behavior (46, 47). Similarly, self-perception of one's health status is also associated with health-promoting lifestyle behaviors (48, 49). Nevertheless, the remaining advanced health literacy skills should still be considered in future studies, as they were associated with work ability in the target group of vocational school students (24).

With respect to sociodemographic factors, sex and educational level did not show relevant associations. Concerning the relationship between sex and work ability, research status appears unclear. As in the present study, mostly no effects are found (50, 51). But there is debate about whether women's multiple roles as employees and familiar caretakers negatively affect their work ability (52). With regard to the educational level, in turn, several studies suggest an effect of educational level on work ability (51–54). Possible reasons for this are that people with a higher level of education more successfully acquire new skills and may have access to greater social and health-related resources leading to a healthier lifestyle (53, 55). In addition, a lower level of education often is associated with demanding physical occupations in which work ability is more likely to be limited (26, 54).

Concerning the effect of age on work ability, however, we found a negative association in the present study. Overall, evidence is heterogenous, but most studies also report a negative association between age and work ability (53, 56, 57). In a German survey, the effect was only observed among women (54). It seems reasonable that work ability declines with age because employees are less able to meet their physical and cognitive work requirements (53). Considering the difference between functional and biological age, a healthy and active lifestyle could have a positive impact on work ability (58) and the negative effect of age can be reduced when health status is taken into account (59).

Nevertheless, it can be assumed, that the study participants‘ health literacy and work ability are also influenced by additional factors apart from the applied model. In a relational understanding of health literacy, it is not only influenced by individual (motivation, competencies, skills) but also by environmental factors (demands, complexity) (60, 61), which is also comparably illustrated by the work ability house model (62). According to this, physical, mental, and social health is the most influential factor apart from working conditions, and therefore considered the basis for work ability (62, 63). Moreover, several studies have shown the relationship between health outcomes and work ability (25, 26, 64) as well as between chronic health problems and early retirement or unemployment (65, 66).

Since the workplace is an important environmental factor for employees' health status, WHP is considered a promising approach for promoting health and work ability (64, 67–70). This has resulted, for example, in WHP being emphasized as a relevant setting for health promotion by the German Prevention Act (6). Overall, about half of the German employees report that WHP is offered in their workplace (71). Considering the WHP offers financed by the German statutory health insurance, 9% of WHP programs addressed older employees and 21% of the WHP offers were targeting people with health-related risk factors in 2020 (72). As our results indicate, these offers address vulnerable groups in terms of health (literacy) and work ability and therefore should be maintained or even expanded and consider individual and environmental conditions as well.

The improvement of health literacy is an overarching core intention of setting-related prevention and health promotion according to the German “Guideline Prevention” (“Leitfaden Prävention”) (73). Thereby, WHP was underlined as an interesting setting for the promotion of health literacy (64, 74). However, theory-based target-group specific approaches in health promotion are rare (75, 76) and currently there is also no consensus about the design of effective interventions in order to promote health literacy (77, 78). In this regard, the structural model of health literacy could serve as an underlying theoretical framework for intervention development and evaluation on the individual level (20). Against the background of a comparatively low participation in WHP offers among people with lower socio-economic status, they could be focused in target-group specific approaches (71).

## Limitations

Firstly, the cross-sectional design does not allow causal conclusions, but the predictive structure of the model seems reasonable. Nevertheless, for intervention evaluation, longitudinal validation is needed. Longitudinal data could indicate whether possible improvements in health literacy skills lead to improved work ability. Additionally, differences in educational level should be considered with regard to their influence on health literacy promotion (27). Secondly, our results cannot be transferred to healthy adults. The identified associations may be target group specific because the inclusion criterion of first health impairments is associated with reduced health literacy (27) and work ability (25, 26, 35). Thirdly, the chosen health literacy questionnaire does not provide cut-off values to appraise participants' health literacy and international comparison is not possible. Until today, there is no gold standard in measuring health literacy due to manifold constructs and definitions (17, 79, 80). Nevertheless, the theoretical foundation of Lenartz's questionnaire is a certain strength (19, 20), so international validation would be beneficial. Finally, regarding PLS-SEM, some possible influential factors on work ability were not analyzed. For example, physical characteristics, health status, leisure-time physical activity, physical and psychosocial work demands, work environment, and social factors do also have an impact on work ability (53, 56, 59, 63). Further studies could include these variables to explain a higher proportion of work ability's variance. Moreover, since currently there is no appropriate model fit criterion in PLS-SEM (81), none was assessed and possible reciprocal or circular mechanisms could not be analyzed with the chosen method (40).

## Conclusion

Lenartz's structural model of health literacy appears to be a valid basis for the development of theory-based health promote on offers for employees with health-related risk factors. Respective interventions should particularly address self-regulation and self-perception in order to promote both, health literacy and work ability. However, additional individual (e.g., health status) and environmental (e.g., work demands) conditions influencing work ability should be considered. Taken together at a general perspective, this study provides an approach for target-group specific analyses and their associations on a health-related construct of interest. The results of such an analysis can be used for the development and evaluation of theory-based interventions in health promotion.

## Data Availability Statement

The datasets used and analyzed during the current study are available from the corresponding author on reasonable request.

## Ethics Statement

The studies involving human participants were reviewed and approved by German Sport University Cologne Ethics Committee. The patients/participants provided their written informed consent to participate in this study.

## Author Contributions

MG and AS: conceptualization. MG and GS: methodology. MG: formal analysis and visualization. MG, GS, and AS: investigation. AS: resources, project administration, and funding acquisition. MG: writing—original draft. GS and AS: writing—review and editing. All authors have read and approved the final manuscript.

## Funding

The AtRisk study is funded by the Federal Ministry of Education and Research (BMBF) (BMBF project number: 01EL1425A) and was a subproject within the research association TRISEARCH.

## Conflict of Interest

The authors declare that the research was conducted in the absence of any commercial or financial relationships that could be construed as a potential conflict of interest.

## Publisher's Note

All claims expressed in this article are solely those of the authors and do not necessarily represent those of their affiliated organizations, or those of the publisher, the editors and the reviewers. Any product that may be evaluated in this article, or claim that may be made by its manufacturer, is not guaranteed or endorsed by the publisher.

## Abbreviations

AVE: average variance extracted
CC: communication and cooperation
CR: composite reliability
DHI: dealing with health information
PAH: proactive approach to health
PLS: partial least squares
SC: self-control
SEM: structural equation modeling
SP: self-perception
SR: self-regulation
WAI: work ability index
WHP: workplace health promotion.

## References

[1] Lampert T Schmidtke C Borgmann L-S Poethko-Müller C Kuntz B. The subjective health of adults in Germany. J Health Monit. (2018) 3:61–8. 10.17886/RKI-GBE-2018-073 PMC8848780 35586373

[2] Heidemann C Scheidt-Nave C Beyer A-K Baumert J Thamm R Maier B. Health situation of adults in Germany–results for selected indicators from GEDA 2019/2020-EHIS. Robert Koch-Institut. (2021) 6:3–25. 10.25646/8459 35146314 PMC8734117

[3] Fuchs J Busch M Lange C Scheidt-Nave C. Prevalence and patterns of morbidity among adults in Germany. Results of the German telephone health interview survey German Health Update (GEDA) 2009. Bundesgesundheitsbl. (2012) 55:576–86. 10.1007/s00103-012-1464-9 22441528

[4] Kieback A Augustin M Heigel H Schäfer I Debus ES. Prävalenz und medikamentöse Therapie des arteriellen Hypertonus der erwerbstätigen Bevölkerung in Deutschland. Gefässchirurgie. (2014) 19:104–8. 10.1007/s00772-013-1268-8

[5] Bundesanstalt für Arbeitsschutz und Arbeitsmedizin (BAuA). Stressreport Deutschland 2019. Berlin; Dortmund; Dresden: Bundesanstalt für Arbeitsschutz und Arbeitsmedizin (BAuA) (2020).

[6] Gesetz zur Stärkung der Gesundheitsförderung und der Prävention: PrävG (2015).

[7] Kickbusch I. Health Literacy. The Solid Facts. Geneva: World Health Organization (2013).

[8] World Health Organization. Promoting Health: Guide to National Implementation of the Shanghai Declaration. Geneva: World Health Organization (2018).

[9] World Health Organization Regional Office for Europe. Health 2020: A European Policy Framework and Strategy for the 21st Century. Geneva: World Health Organization (2013).

[10] Commission of the European Communities. Together for Health: A Strategic Approach For the EU 2008-2013. Brussels (2007). Available online at: https://ec.europa.eu/commission/presscorner/detail/en/IP_07_1571 (accessed October 23, 2007).

[11] World Health Organization. Ottawa Charter for Health Promotion (1986).

[12] Sørensen K van den Broucke S Fullam J Doyle G Pelikan J Slonska Z. Health literacy and public health: a systematic review and integration of definitions and models. BMC Public Health. (2012) 12:1–13. 10.1186/1471-2458-12-80 22276600 PMC3292515

[13] Sørensen K Pelikan JM Röthlin F Ganahl K Slonska Z Doyle G. Health literacy in Europe: comparative results of the European health literacy survey (HLS-EU). Eur J Public Health. (2015) 25:1053–8. 10.1093/eurpub/ckv043 25843827 PMC4668324

[14] Jordan S Hoebel J. Gesundheitskompetenz von Erwachsenen in Deutschland : Ergebnisse der Studie “Gesundheit in Deutschland aktuell” (GEDA). Bundesgesundheitsblatt Gesundheitsforschung Gesundheitsschutz. (2015) 58:942–50. 10.1007/s00103-015-2200-z 26227894

[15] Berkman ND Davis TC McCormack L. Health literacy: what is it? J Health Commun. (2010) 15 Suppl 2:9–19. 10.1080/10810730.2010.499985 20845189

[16] DeWalt DA Hink A. Health literacy and child health outcomes: a systematic review of the literature. Pediatrics. (2009) 124 Suppl 3:S265–74. 10.1542/peds.2009-1162B 19861480

[17] Bitzer EM Sørensen K. Gesundheitskompetenz–health literacy. Gesundheitswesen. (2018) 80:754–66. 10.1055/a-0664-0395 30176683

[18] Malloy-Weir LJ Charles C Gafni A Entwistle V. A review of health literacy: Definitions, interpretations, and implications for policy initiatives. J Public Health Policy. (2016) 37:334–52. 10.1057/jphp.2016.18 27193502

[19] Lenartz N. Gesundheitskompetenz und Selbstregulation. Göttingen: V&R unipress University Press (2012).

[20] Soellner R Lenartz N Rudinger G. Concept mapping as an approach for expert-guided model building: the example of health literacy. Eval Program Plann. (2017) 60:245–53. 10.1016/j.evalprogplan.2016.10.007 27771012

[21] Soellner R Huber S Lenartz N Rudinger G. Facetten der Gesundheitskompetenz–eine Expertenbefragung. Projekt Gesundheitskompetenz. In: Klieme E Leutner D Kenk M editors. Kompetenzmodellierung. Zwischenbilanz des DFG-Schwerpunktprogramms und Perspektiven des Forschungsansatzes. Weinheim: Beltz (2010). p. 104–114. 10.25656/01:3384

[22] Kuhlmann K Beauducel A Predel G Preuß M Preuß P Rudinger G. Evaluation des Gesundheitsverhaltens Studierender. Diagnostica. (2015) 61:163–71. 10.1026/0012-1924/a00014328686276

[23] Fiedler S Pfaff H Soellner R Pförtner T-K. Exploring the association between health literacy and psychological well-being among industry managers in Germany. J Occup Environ Med. (2018) 60:743–53. 10.1097/JOM.0000000000001324 29557837

[24] Stassen G Grieben C Hottenrott N Rudolf K Froböse I Schaller A. Associations between health-related skills and young adults' work ability within a structural health literacy model. Health Promot Int. (2020) 36:1072–83. 10.1093/heapro/daaa099 33319224 PMC8405247

[25] Bethge M Spanier K Peters E Michel E Radoschewski M. Self-reported work ability predicts rehabilitation measures, disability pensions, other welfare benefits, and work participation: longitudinal findings from a sample of German employees. J Occup Rehabil. (2018) 28:495–503. 10.1007/s10926-017-9733-y 28956225

[26] Gould R Ilmarinen J Järvisalo J Koskiinen S. Dimensions of Work Ability: Results of the Health 2000 Survey. Helsinki: Finnish Centre for Pension (2008).

[27] Schaeffer D Berens E-M Gille S Griese L Klinger J de Sombre S. Gesundheitskompetenz der Bevölkerung in Deutschland vor und während der Corona Pandemie: Ergebnisse des HLS-GER 2. Universität Bielefeld, Interdisziplinäres Zentrum für Gesundheitskompetenzforschung (2021).

[28] Lampert T Kroll L Kuntz B Hoebel J. Health inequalities in Germany and in international comparison: trends and developments over time. J Health Monit. (2018) 3:1–24. 10.17886/RKI-GBE-2018-036 PMC8864567 35586261

[29] Schaller A Dejonghe L Alayli-Goebbels A Biallas B Froboese I. Promoting physical activity and health literacy: study protocol for a longitudinal, mixed methods evaluation of a cross-provider workplace-related intervention in Germany (The AtRisk study). BMC Public Health. (2016) 16:626. 10.1186/s12889-016-3284-6 27449188 PMC4957840

[30] Hasselhorn HM Freude G. Der Work Ability Index: Ein Leitfaden. Bremerhaven: Wirtschaftsverl NW Verl für neue Wiss (2007).

[31] Thinschmidt M Seibt R. “Work Ability-Index”—Vergleich von Lang- und Kurzversion der Krankheitsdiagnosen anhand einer deutschen Stichprobe. Zbl Arbeitsmed. (2007) 57:212–21. 10.1007/BF03349124

[32] Radkiewicz P Widersal-Bazyl M The The NEXT-Study group. Psychometric properties of Work Ability Index in the light of comparative survey study. Int Congr Ser. (2005) 1280:304–9. 10.1016/j.ics.2005.02.089

[33] Jääskeläinen A Kausto J Seitsamo J Ojajärvi A Nygård CH Arjas E. Work ability index and perceived work ability as predictors of disability pension: a prospective study among Finnish municipal employees. Scand J Work Environ Health. (2016) 42:490–9. 10.5271/sjweh.3598 27706492

[34] Lundin A Leijon O Vaez M Hallgren M Torgén M. Predictive validity of the work ability index and its individual items in the general population. Scand J Public Health. (2017) 45:350–6. 10.1177/1403494817702759 28385066

[35] Bethge M Radoschewski FM Gutenbrunner C. The work ability index as a screening tool to identify the need for rehabilitation: longitudinal findings from the second German sociomedical panel of employees. J Rehabil Med. (2012) 44:980–7. 10.2340/16501977-1063 23027375

[36] Zwart BCH de Frings-Dresen MHW van Duivenbooden JC. Test-retest reliability of the Work Ability Index questionnaire. Occup Med. (2002) 52:177–81. 10.1093/occmed/52.4.177 12091582

[37] Little RJA. A test of missing completely at random for multivariate data with missing values. J Am Stat Assoc. (1988) 83:1198–202. 10.1080/01621459.1988.10478722

[38] Ringle CM Wende S Will A. SmartPLS 2.0 (beta) Hamburg (2005).

[39] Rigdon EE. Rethinking partial least squares path modeling: in praise of simple methods. Long Range Plann. (2012) 45:341–58. 10.1016/j.lrp.2012.09.010

[40] Hair JF Hult GTM Ringle CM Sarstedt M. A primer on partial least squares structural equation modeling (PLS-SEM) Second edition. Los Angeles, London, New Delhi, Singapore, Washington DC, Melbourne: SAGE (2017).

[41] Fornell C Larcker DF. Evaluating Structural Equation Models with Unobservable Variables and Measurement Error. J Mark Res. (1981) 18:39. 10.2307/3151312

[42] Cohen J. Statistical Power Analysis for the behavioral sciences. Burlington: Elsevier Science (2013).

[43] Jahn S. Strukturgleichungsmodellierung mit LISREL, AMOS und SmartPLS: Eine Einführung (An Introduction to Structural Equation Modeling with LISREL, AMOS and SmartPLS). SSRN Journal. (2007). Available online at: https://ssrn.com/abstract=2729658 10.2139/ssrn.2729658

[44] Barclay D Higgins C Thompson R. The partial least squares (PLS) approach to casual modeling: personal computer adoption and use as an Illustration. Technol Stud Spec Issue Res Methodol. (1995) 2:285–309.

[45] Kwasnicka D Dombrowski SU White M Sniehotta F. Theoretical explanations for maintenance of behaviour change: a systematic review of behaviour theories. Health Psychol Rev. (2016) 10:277–96. 10.1080/17437199.2016.1151372 26854092 PMC4975085

[46] Anderson ES Winett RA Wojcik JR. Self-regulation, self-efficacy, outcome expectations, and social support: social cognitive theory and nutrition behavior. Ann Behav Med. (2007) 34:304–12. 10.1007/BF02874555 18020940

[47] Shieh C Weaver MT Hanna KM Newsome K Mogos M. Association of self-efficacy and self-regulation with nutrition and exercise behaviors in a community sample of adults. J Community Health Nurs. (2015) 32:199–211. 10.1080/07370016.2015.1087262 26529105

[48] Silva AOd Diniz PRB Santos MEP Ritti-Dias RM Farah BQ Tassitano RM. Health self-perception and its association with physical activity and nutritional status in adolescents. J Pediatr. (2019) 95:458–65. 10.1016/j.jped.2018.05.007 29957248

[49] Robinson E Haynes A Sutin A Daly M. Self-perception of overweight and obesity: a review of mental and physical health outcomes. Obes Sci Pract. (2020) 6:552–61. 10.1002/osp4.424 33082997 PMC7556430

[50] Martinez MC. Latorre MdRDdO. Saúde e capacidade para o trabalho em trabalhadores de área administrative. Rev Saude Publica. (2006) 40:851–8. 10.1590/S0034-89102006000600015 17301907

[51] Monteiro MS Ilmarinen J Corrâa Filho HR. Work ability of workers in different age groups in a public health institution in Brazil. Int J Occup Saf Ergon. (2006) 12:417–27. 10.1080/10803548.2006.11076703 17156617

[52] Godinho MR Greco RM Teixeira MTB Teixeira LR Guerra MR Chaoubah A. Work ability and associated factors of Brazilian technical-administrative workers in education. BMC Res Notes. (2016) 9:1–10. 10.1186/s13104-015-1837-x 26725043 PMC4698321

[53] Yang T Liu T Lei R Deng J Xu G. Effect of Stress on the Work Ability of Aging American Workers: Mediating Effects of Health. Int J Environ Res Public Health. (2019) 16:2273. 10.3390/ijerph16132273 31252597 PMC6650795

[54] Bethge M Radoschewski FM Müller-Fahrnow W. Work stress and work ability: cross-sectional findings from the German sociomedical panel of employees. Disabil Rehabil. (2009) 31:1692–9. 10.1080/09638280902751949 19479539

[55] Robert Koch-Institut. Gesundheitliche Ungleichheit in Deutschland und im internationalen Vergleich: Zeitliche Entwicklungen und Trends. RKI-Bib1 (Robert Koch-Institut) (2018).

[56] van den Berg TIJ Elders LAM Zwart BCH de Burdorf A. The effects of work-related and individual factors on the Work Ability Index: a systematic review. Occup Environ Med. (2009) 66:211–20. 10.1136/oem.2008.039883 19017690

[57] Safari S Akbari J Kazemi M Mououdi MA Mahaki B. Personnel's health surveillance at work: effect of age, body mass index, and shift work on mental workload and work ability index. J Environ Public Health. (2013) 2013:289498. 10.1155/2013/289498 23956756 PMC3730146

[58] Kenny GP Yardley JE Martineau L Jay O. Physical work capacity in older adults: implications for the aging worker. Am J Ind Med. (2008) 51:610–25. 10.1002/ajim.20600 18543279

[59] McGonagle AK Barnes-Farrell JL Di Milia L Fischer FM Hobbs BBB Iskra-Golec I. Demands, resources, and work ability: a cross-national examination of health care workers. Eur J Work Organ Psychol. (2014) 23:830–46. 10.1080/1359432X.2013.819158

[60] Parker R. Measuring health literacy: What? So what? Now what In: Measures of Health Literacy: Workshop Summary. Washington DC: National Academies Press (2009).

[61] Parker R Ratzan SC. Health literacy: a second decade of distinction for Americans. J Health Commun. (2010) 15 Suppl 2:20–33. 10.1080/10810730.2010.501094 20845190

[62] Tempel J Ilmarinen J editors. Arbeitsleben 2025: Das Haus der Arbeitsfähigkeit im Unternehmen bauen. Hamburg: VSA-Verl. (2013).

[63] Ilmarinen J Tuomi K Seitsamo J. New dimensions of work ability. Int Congr Ser. (2005) 1280:3–7. 10.1016/j.ics.2005.02.060

[64] Hamacher W Eickholt C Lenartz N Blanco Trillo S. Sicherheits-und Gesundheitskompetenz durch informelles Lernen im Prozess der Arbeit. Berlin; Dortmund; Dresden: Bundesanstalt für Arbeitsschutz und Arbeitsmedizin (2012).

[65] Fleischmann M Carr E Stansfeld SA Xue B Head J. Can favourable psychosocial working conditions in midlife moderate the risk of work exit for chronically ill workers? A 20-year follow-up of the Whitehall II study. Occup Environ Med. (2018) 75:183–90. 10.1136/oemed-2017-104452 29042407 PMC5869452

[66] Leijten FRM Wind A de van den Heuvel SG Ybema JF van der Beek AJ Robroek SJW. The influence of chronic health problems and work-related factors on loss of paid employment among older workers. J Epidemiol Community Health. (2015) 69:1058–65. 10.1136/jech-2015-205719 26112957

[67] Oakman J Neupane S Proper KI Kinsman N Nygård C-H. Workplace interventions to improve work ability: a systematic review and meta-analysis of their effectiveness. Scand J Work Environ Health. (2018) 44:134–46. 10.5271/sjweh.3685 29493713

[68] Lusa S Punakallio A Mänttäri S Korkiakangas E Oksa J Oksanen T. Interventions to promote work ability by increasing sedentary workers' physical activity at workplaces-a scoping review. Appl Ergon. (2020) 82:102962. 10.1016/j.apergo.2019.102962 31568961

[69] Luxembourg Declaration on Workplace Health Promotion in the European Union. Available online at: https://www.enwhp.org/resources/toolip/doc/2018/04/24/luxembourg_declaration.pdf

[70] Barthelmes I Bödeker W Sörensen J Kleinlercher K Odoy J. Wirksamkeit und Nutzen arbeitsweltbezogener Gesundheitsförderung und Prävention. Zusammenstellung der wissenschaftlichen Evidenz 2012 bis 2018. Dresden (2019).

[71] Hollederer A. Betriebliche Gesundheitsförderung in Deutschland für alle? Ergebnisse der BIBB-/BAuA-Erwerbstätigenbefragung 2018. Gesundheitswesen (2021). 10.1055/a-1658-0125 PMC10125318 34758504

[72] Schempp N Römer K Medizinischer Dienst des Spitzenverbandes Bund der Krankenkassen e. V. (MDS). Präventionsbericht 2021: Leistungen der gesetzlichen Krankenversicherung: Primärprävention und Gesundheitsförderung. Leistungen der sozialen Pflegeversicherung: Prävention in stationären Pflegeeinrichtungen. Berichtsjahr 2020. Berlin (2021).

[73] GKV Spitzenverband. Leitfaden Prävention– Handlungsfelder und Kriterien nach §20 Abs.2 SGBV. Berlin (2021).

[74] Kickbusch I Maag D Kris H. Health Literacy. International Encyclopedia of Public Health. 2008: 204–11.

[75] Dalgetty R Miller CB Dombrowski SU. Examining the theory-effectiveness hypothesis: A systematic review of systematic reviews. Br J Health Psychol. (2019) 24:334–56. 10.1111/bjhp.12356 30793445

[76] Walters R Leslie SJ Polson R Cusack T Gorely T. Establishing the efficacy of interventions to improve health literacy and health behaviours: a systematic review. BMC Public Health. (2020) 20:1040. 10.1186/s12889-020-08991-0 32605608 PMC7329558

[77] Manafo E Wong S. Health literacy programs for older adults: a systematic literature review. Health Educ Res. (2012) 27:947–60. 10.1093/her/cys067 22752153

[78] Brainard J Wilsher SH Salter C Loke YK. Methodological review: quality of randomized controlled trials in health literacy. BMC Health Serv Res. (2016) 16:246. 10.1186/s12913-016-1479-2 27402048 PMC4940982

[79] Altin SV Finke I Kautz-Freimuth S Stock S. The evolution of health literacy assessment tools: a systematic review. BMC Public Health. (2014) 14:1207. 10.1186/1471-2458-14-1207 25418011 PMC4289240

[80] Haun JN Valerio MA McCormack LA Sørensen K Paasche-Orlow MK. Health literacy measurement: an inventory and descriptive summary of 51 instruments. J Health Commun. (2014) 19(Suppl 2):302–33. 10.1080/10810730.2014.936571 25315600

[81] Henseler J Sarstedt M. Goodness-of-fit indices for partial least squares path modeling. Comput Stat. (2013) 28:565–80. 10.1007/s00180-012-0317-1

